# Granting access: Development of a formal course to demystify and promote predoctoral fellowship applications for graduate students

**DOI:** 10.1371/journal.pone.0301480

**Published:** 2024-04-26

**Authors:** Pamela K. Geyer, Darren S. Hoffmann, Jennifer Y. Barr, Heather A. Widmayer, Christine M. Blaumueller

**Affiliations:** 1 Department of Biochemistry and Molecular Biology, University of Iowa, Iowa City, Iowa, United States of America; 2 Medical Scientist Training Program, University of Iowa, Iowa City, Iowa, United States of America; 3 Department of Anatomy and Cell Biology, University of Iowa, Iowa City, Iowa, United States of America; 4 Scientific Editing and Research Communication Core, Carver College of Medicine, University of Iowa, Iowa City, Iowa, United States of America; Massachusetts Institute of Technology School of Engineering, UNITED STATES

## Abstract

Strong scientific writing skills are the foundation of a successful research career and require training and practice. Although these skills are critical for completing a PhD, most students receive little formal writing instruction prior to joining a graduate program. In 2015, the University of Iowa Medical Scientist Training Program (MSTP) addressed this issue by developing the scientific writing course Grant Writing Basics (GWB). Here we describe the structure of this course and its effectiveness. GWB is an interactive, workshop-based course that uses a National Institutes of Health (NIH) F30 predoctoral fellowship proposal as a platform for building writing expertise. GWB incorporates established pedagogical principles of adult learning, including flipped classrooms, peer teaching, and reiterative evaluation. Time spent in class centers on active student analysis of previously submitted fellowship applications, discussion of writing resources, active writing, facilitated small group discussion of critiques of student writing samples, revision, and a discussion with a panel of experienced study section members and a student who completed a fellowship submission. Outcomes of GWB include a substantial increase in the number of applications submitted and fellowships awarded. Rigorous evaluation provides evidence that learning objectives were met and that students gained confidence in both their scientific writing skills and their ability to give constructive feedback. Our findings show that investment in formal training in written scientific communication provides a foundation for good writing habits, and the knowledge and skills needed to succeed in this vital aspect of a scientific research career. Furthermore, they highlight that evaluation is valuable in guiding course evolution. Strategies embedded in GWB can be adapted for use in any graduate program to advance scientific writing skills among its trainees.

## Introduction

Effective written communication is central to success in a scientific career. Scientists must share their research findings to garner funds and build successful research teams. Yet, writing scientific papers and grant proposals is inherently complex and requires considerable time and effort. Self-reported barriers to engagement in scientific writing, especially for early-career scientists, include lack of sufficient time and aptitude [1].

Effective writing is shaped by instruction and practice. Although such training should begin early in a scientific career, most undergraduate curricula include little formal instruction in scientific writing [2]. This deficit reflects the attitude that time spent learning writing skills is less important than time spent learning foundational scientific concepts. As a result, students enter graduate programs with limited practice in scientific writing, a core competency of PhD training programs [3]. This gap continues during graduate training, evidenced by a recent survey of MD-PhD programs revealing that few programs include writing courses in their curricula [4]. Indeed, in a recent survey of alumni of a molecular medicine PhD program, participants ranked “proficiency in academic and professional writing” as very important (4/4) for career success but ranked satisfaction with their preparation lower (3/4; [5]). These observations about gaps in training highlight a need for dedicated coursework in scientific writing.

Recognizing the importance of writing in scientific careers, the leadership of the National Institutes of Health (NIH)-funded University of Iowa Medical Scientist Training Program (MSTP) developed a course entitled Grant Writing Basics (GWB) to augment training of their MD-PhD dual-degree students, a cohort of talented trainees whose career goals include advancing the science of medicine and its translation into clinical practice. GWB uses the NIH F30 predoctoral fellowship application as a platform for instruction, given that this type of application is a relevant exercise, is a likely next step for many MSTP trainees, and represents an authentic learning experience. We reasoned that early participation in GWB would motivate students to develop key questions pertinent to their research interests, boost the amount of concentrated time that they spend on reading and critically analyzing the literature, increase their understanding of experimental design, and fine-tune their research goals. Additionally, students would practice delivering well-reasoned and concise scientific arguments to a knowledgeable but broad peer audience, building upon concepts that peer learning increases engagement and enhances knowledge [6]. These design choices align with principles of adult learning, including relevance, self-direction, ownership, and learning by doing [7]. Finally, submission of a fellowship proposal should provide students the opportunity to understand the mechanics of, and time commitments required for, the grant writing process, as well provide eligibility for institutional monetary recognition. GWB occurs early during the PhD training and helps establish a foundation for continued formal and informal writing instruction as students advance through their PhD programs.

The University of Iowa MSTP is not alone in adopting grant writing as a platform for instruction of scientific writing. Surveys of the landscape of grant writing instruction reveal that multiple formats are used, ranging from focused singular workshops to longitudinal courses and writing communities [4,8–11]. Common instructional features include identifying funding sources, decoding grant-specific language (e.g., specific aims, training plans), introducing students to the mechanics of the grant review process, and providing dedicated time for iterative writing that is focused on the application [9,11]. Notably, each grant writing course is highly contextualized, and built to suit the student population and grant writing norms of the discipline.

Here, we describe key aspects of GWB, including the evaluation process and our findings that resulted in the current course structure. We report that students have gained confidence in their grantsmanship and scientific writing abilities, have become more willing to seek feedback on their own work and are more assured about providing useful feedback to others, skills with long-lasting benefits. Our findings also highlight the value of rigorous evaluation in guiding iterative evolution of curricula. Together, our observations provide strong support for early training in written scientific communication.

## Methods

### Course participants

GWB is a one-credit course that is required for all third year MSTP students. This course was developed with the goals of building a learner’s autonomy, encouraging active learning experiences, inspiring ownership, and providing the structure needed to achieve learning objectives. Embedded in GWB are milestones that mark students’ progress, and opportunities for engagement as peer educators. These design features align with principles outlined in adult learning theory [7,12,13].

Each year, the course is taken by six to eleven students who are members of a single MSTP entering class. MSTP students have strong scientific backgrounds. In recent MSTP entering classes (2015 to 2019), most (~75%) students had completed one or more “gap years” during which they engaged in scientific research, and over half (55%) were co-authors on at least one paper. MSTP students have diverse scientific interests, evidenced by their enrollment in one of fifteen PhD programs across three colleges (Table 1). The MSTP class composition has two major advantages. First, students have a shared history of training that provides the social capital and trust needed for giving and receiving honest and productive feedback. Second, given the diverse nature of research interests, the students are forced to write to a broad audience, in language that is clear and concise.

**Table 1:** Demographics of GWB participants 2015 to 2021.

College	Graduate program	# Students	# Submissions	# Funded
Medicine	Biochemistry	2	1	1
	Cancer Biology	3	3	2
	Cell and Developmental Biology	1	1	0
	Free Radical Radiation Biology	1	1	1
	Genetics	7	5	3
	Immunology	9	6	3
	Microbiology	4	3	1
	Molecular Medicine	10	9	5
	Molecular Physiology and Biophysics	7	7	3
	Neuroscience	11	8	3
	Pharmacology	1	1	1
	Medicine	56	45 (80%)	23 (51%)
Engineering	Biomedical Engineering	4	3	0
	Chemical and Biochemical Engineering	1	1	1
	Engineering	5	4 (80%)	1 (25%)
Public Health	Biostats	2	0	0
	Epidemiology	3	2	1
	Public Health	5	2 (40%)	1 (50%)
	Total	66	51 (77%)	25 (49%)

n = 66.

### Course description

GWB uses the NIH predoctoral F30 National Research Service Award (a funding mechanism specific to physician-scientists in training) as a platform for building writing expertise. Students take GWB in the spring semester of their first year of graduate training, after having completed two years of preclinical medical school requirements. At this stage, students have identified a research mentor, and most have begun research relevant to their dissertation topic. The third year of the MSTP is equivalent to the second semester of the second year of graduate training for most PhD students. The objectives of GWB are: 1) to learn the scientific logic that underlies effective grant writing; 2) to learn the value of revision; 3) to develop skills in providing and applying constructive feedback; and 4) to assume ownership of a research project. An additional expectation is that students will gain a better understanding of how to assemble a predoctoral fellowship. An unofficial expectation is that students will submit a full proposal for extramural funding. The final GWB writing product is a polished Specific Aims page that serves as a roadmap for a fellowship submission, and ideally for the student’s dissertation research.

GWB is taught by a team of instructors who integrate expertise in scientific investigation and communication. This team includes a member of the MSTP PhD training faculty (PKG) and scientists (henceforth referred to as “editors”) on the staff of the Scientific Editing and Research Communication Core in the Carver College of Medicine (CMB, JYB, HAW). We have found that this combination of trainers is highly effective, with the faculty member putting greater emphasis on scientific issues and the editors additionally providing substantive advice on effective writing strategies.

### Course format

Weekly course sessions are one-and-a-half hours in length and are held over a three-month semester (11 weeks). Classes are interactive, workshop based, and delivered in four formats, as indicated in Table 2. Five sessions are held in a large group setting and cover foundational material related to writing components of the F30 application and strategies for clarity in writing; four sessions are held in a small group setting (writing groups) and focus on peer driven critiques of Specific Aims pages submitted by the students; one session combines small and large group formats to help students sharpen the logic underlying their proposal; and one session is a panel discussion that features faculty who have reviewed fellowship applications as members of study sections and an MSTP student who has submitted a fellowship application.

**Table 2:** Course sessions for GWB.

Week	Topic	Session Format
	Pre-course Interview	
1	Specific Aims (SA) page	Workshop, large group
2	The logic of the scientific story	Workshop, partner/large group
3	Achieving clarity in writing	Workshop, large group
4	The Research Strategy/ rigor and reproducibility	Workshop, large group
5	The Biographical Sketch	Workshop, large group
6	Critiques of SA pages	Critique, small writing group
7	Critiques of SA pages	Critique, small writing group
8	Documents relevant to the Training Plan	Workshop, large group
9	Study section panel	Panel discussion
10	Critiques of SA pages	Critique, small writing group
11	Critiques of SA pages	Critique, small writing group
	Post-course Interview	

The five course sessions that cover foundational material are delivered using a flipped-classroom model, a teaching modality found to increase students’ motivation and engagement in learning [14]. Self-directed student learning includes review of pre-recorded lectures and two key resources: grant writing templates and examples of grant sections. Templates contain prompts and guidelines for each section of the fellowship and were developed by the editors based on NIH resources, the editors’ own experiences, and well-regarded grant writing workbooks [15,16]. Example texts are excerpts from previous NIH-funded fellowship proposals that either model effective scientific writing or could use improvement. Students are expected to be prepared to participate in a facilitated discussion of lessons learned from these examples and the lecture materials. Each session includes about one hour of group discussion on topics such as the Specific Aims page, Research Strategy, Biographical Sketch, or Training Plan (Table 2) and thirty minutes during which students brainstorm and begin to write a section of the grant using the relevant template. Each session ends with a brief wrap-up discussion.

The four course sessions devoted to critiquing Specific Aims pages use a small group format with a high degree of structure that enables rounds of feedback from diverse sources. Each student submits a draft for two of these sessions. In an early session, they submit an initial draft, and in a follow up session, they submit a revised draft that addresses feedback received during the earlier discussion. Groups include one facilitator (an editor) and three to four students. Early in the course, students are introduced to concepts of giving and receiving feedback [17], and instructors emphasize the value of, and strategies for, doing so effectively. Examples of constructive feedback are provided to guide the written critiques the students prepare. They submit their draft of the Specific Aims page to members of their writing group (including the facilitator) and the faculty instructor one week before it is discussed. Writers are encouraged to include a letter outlining areas on which they wish to obtain feedback. Each Specific Aims page is pre-assigned to a primary reviewer, who provides detailed written and verbal feedback; the remaining students and facilitator also provide written and verbal commentary. Feedback from other students is not expected to be as detailed as that of the primary reviewer; feedback from the instructors is typically thorough. During the session, the primary reviewer introduces the proposed research project to the group and then summarizes the strengths of the writing and areas needing improvement. Next, other student reviewers contribute their ideas, and finally the facilitator provides comments and synthesizes the collective feedback. Students also receive written feedback from the faculty instructor. During the review of each Specific Aims page, the writer is present but initially silent; after the review is completed, the writer is invited to ask questions and share ideas for addressing the issues that were raised. Setting boundaries for the participation of the writer allows reviewers to complete their assessment of the document without the discussion digressing into the author’s intended meaning. The small size of the writing group ensures that the feedback provided to writers is manageable and comes from trusted peers and a facilitator. This approach fosters productive discussions. A revised version of the Specific Aims page is reviewed several weeks later, following the same structured format. In this session, discussion includes an assessment of how effectively the feedback from the first session was addressed.

Early in GWB, a single session is devoted to an exercise in refining the logic of the students’ research proposal through a storytelling approach [18]. This session is a supplement to the foundational presentation about the Specific Aims page, which is held the previous week (Table 2). During this exercise, students are asked to think about the current status of their field of study, how they envision the field will advance in the future, and how their proposed research will promote change. Students are initially paired with a partner for a ten-minute discussion during which each presents their story to the other, switching off as “storyteller” and “reporter.” The large group then reconvenes, and each reporter retells the story they heard. In many cases, this exercise reveals logic gaps that the storyteller needs to fill when writing their Specific Aims page. This teaching exercise builds on what the students have already learned about writing a Specific Aims page, by requiring them to consider the logic underlying their proposal from a new angle and from a listener’s (and reader’s) perspective. This session has proven to be particularly helpful in refining the Specific Aims page, and it has helped students start thinking about the Significance section of the Research Strategy.

Midway through GWB, a single course session is devoted to a panel discussion. This session occurs in a large group setting and is facilitated by the faculty instructor. The panel consists of three to four faculty members who have served on predoctoral review panels for the NIH or private foundations and one senior MSTP student who has experience submitting a fellowship application. The panel session is held after students have been introduced to all sections of the fellowship application and have had the first draft of their Specific Aims page critiqued. This discussion provides insights into the score-driving components of a proposal, from both a faculty member’s and a student’s perspective. Notably, the panel discussion highlights that grant evaluation is subjective and reviewer-dependent; this helps overcome a commonly held misperception that reviews are necessarily objective. In addition, panelists share information about local resources and real-life experiences that have helped them develop successful proposals.

### Evaluations of the GWB course

Evaluation procedures in this study included anonymous electronic course surveys and pre- and post-course interviews that were conducted by the editors and broadly evaluated a student’s experience. Evaluations were completed by students in nine classes of GWB students between 2015 and 2023. All interview notes and survey data were anonymized.

In years 2015 and 2016 (n = 19 students), course evaluation was conducted in class, during the final course session. In years 2017 to 2023 (n = 63 students), anonymous electronic surveys were administered at the end of the course. Survey questions were answered using a five-point Likert agreement scale. Scaled response items are summarized in Table 3. In addition, three open field questions were asked, including 1) What strategies do you find most useful? 2) What changes might improve the templates? and 3) Have you overcome problems that you previously experienced in writing? The response rate for electronic course evaluations was 97% (61/63).

**Table 3:** Outcomes of anonymous electronic course evaluations for 2017 to 2023.

Query	Percent				
	Strongly Disagree	Disagree	Neutral	Agree	Strongly Agree
Grant knowledge increased	0	3	2	44	51
Specific Aims page template was useful	0	2	6	44	48
Feedback from peers was useful	0	5	7	25	63
Providing feedback to others helped me	0	2	7	40	51
Ability to provide feedback improved	2	2	13	47	36
Feel prepared to write a fellowship	2	5	16	33	44

n = 61 of 63 (97% response rate).

In years 2020 to 2023 (n = 32 students), pre- and post-course interviews were conducted by the team of editors, who interviewed each individual student. During the interviews, students were reminded that responses would be made confidential and held no weight in their standing in the program. Notably, the interviewers did not hold an ongoing authoritative role with the students, reducing impacts of perceived power dynamics. An open-question format was used to develop an understanding of students’ experience, in their own words. Pre-course interviews enabled the instructional team to informally assess student expectations and experiences, and to develop trust that promoted honest class discussions. Post-course interviews enabled a deeper exploration of the extent to which GWB fostered knowledge and skill development in grant writing, areas for continued personal growth, and other course outcomes. Post-course interview questions touched on strengths of the course format and areas for improvement. One member of the interview team (CMB) took detailed notes during the interviews. After the interview, notes were reviewed by the interview team to fill any gaps that were missed. Then, the interview note taker determined the frequency of responses to establish initial response codes. Subsequently, the MSTP program evaluator (DSH) conducted a thematic analysis of consolidated data that involved clustering themes into categories and re-analyzing the data in a focused coding step. Pre- and post-course interview scripts are provided in S1 Appendix.

### Ethics statement

The University of Iowa institutional review board determined that the evaluation of GWB impact did not meet the regulatory definition of human subjects research after review (IRB #202302103).

## Results

### Electronic evaluations and changes to course structure

Anonymous electronic course evaluations revealed enthusiastic endorsement of GWB (Table 3). Of the 63 students surveyed, most (95%) agreed that their knowledge of how to write a grant proposal had improved. Most (92%) also found that the grant-writing templates developed by the editing core were useful, with answers to free-form questions indicating that these resources effectively prompted development of sections of the proposal. For example, students indicated that the template for the Specific Aims page “Helps with blank page anxiety” and “Provides a place to get started,” endorsing the use of grant writing templates in aiding the writing process.

A cornerstone of GWB is the use of peer driven feedback and revision to develop a polished Specific Aims page. Electronic evaluations indicated that most students found receiving (88%) and giving (91%) feedback was useful, even though fewer (83%) reported satisfaction with their skills in providing feedback. Written comments included statements such as, “Having the requirement to write and revise in this course greatly improved the outcome of my writing.” These observations support the inclusion of opportunities for revision in any scientific writing course.

Early evaluations revealed that some structural aspects of the course were not effective (see S1 Table for additional details). One issue related to the timing of GWB sessions. Initially, one-hour sessions were held twice per week over a one-and-a-half-month period. When queried, most participants (58%) preferred holding longer sessions over a semester (three months). The course was adapted accordingly. A second issue concerned the delivery of course content. Comments included “Lectures were good, but we got the information all at the same time, with no active participation component, so I retained very little of the lecture” and “A webinar style lecture where we look at some of those examples or PowerPoints at home with a recorded lecture would allow us to spend more time writing and revising.” In response, the GWB format was changed to a fully flipped-classroom style [14]; this allows for more extensive discussion and inclusion of a short in-class writing period, advancing alignment of GWB with adult learning theory [7,12]. A third issue centered on critique sessions. During the early years, these sessions included all eight to eleven students plus the instructors. Course evaluations revealed that the amount of feedback received was overwhelming and that some students were confused by inconsistencies in feedback. As summarized by one student, “Honestly, I didn’t look at everyone’s reviews because it was just too much for me to consider.” Based on these perspectives, small writing groups were adopted, and the composition of each group remained constant throughout the semester. This gave students a better sense of the strides they had made through the revision process. A fourth concern, voiced by nearly a third of students, was that they did not feel prepared to submit the full proposal. Indeed, in recent evaluations, several respondents stated that they wished the course was longer so that they could receive feedback on every section of the proposal. Whereas the GWB instructors appreciated these views, they recognized that such a labor-intensive approach would be untenable, requiring more time than members of the instructional team could commit to this course. Instructors share the opinion that writing a Specific Aims page effectively illustrates principles of scientific writing. As such, the critique sessions have remained focused on this section of a fellowship proposal. Nonetheless, this feedback motivated the team to provide class time for students to brainstorm and/or write individual sections; this adaptation was intended to meet the students halfway.

### The value of pre- and post-course interviews

Themes from the interviews are summarized in Table 4. Pre-course interviews revealed that many students had little to no grant writing experience prior to GWB. Further, their broader experience with scientific writing was often restricted to writing a section of a manuscript. When students were asked to identify writing strengths, the responses fell into three thematic categories: management of the writing process; skills with technical language; and scientific literacy (most commonly expressed as being able to communicate the broader picture of their scientific research). When students were asked to identify writing weaknesses, the most frequent response was productivity practices (e.g., outlining, organizing, drafting). Self-described areas for improvement fell into four clusters: technical language skills; management of the writing process (organization of the writing process); inner dynamics of writing (e.g., internal criticism, self-doubt, spending too long in planning mode); and scientific literacy (thinking broadly about their science). These improvement areas were strongly aligned with the objectives of GWB.

**Table 4:** Outcomes of pre- and post-course interviews of course participants from 2020 to 2023.

Pre-Course		Post-Course	
STRENGTHS			
*Themes*	*Examples*	*Themes*	*Examples*
Management of Writing Process	Outlining Organization Drafting Finding flow	New Structured Writing Strategies	Scheduling time Defining task lists Using templates Defining the big picture Drafting in bullet points
Technical Language Skills	Conciseness Creativity Transitions Syntax	Technical Language Skills	More concise and clear Sentence structure/mechanics
Scientific Literacy	Big picture Critical reading	Feedback	Increased desire for feedback Increased skill in giving and receiving feedback
IMPROVEMENT AREAS			
*Themes*	*Examples*	*Themes*	*Examples*
Technical Language Skills	Conciseness Flow in paragraphs Simplistic voice	Technical Language Skills	Clarity Detail and word count Creativity and voice Grammar/syntax
Management of Writing Process	Sequenced goals Outline first writing	Management of Writing Process	Managing time and process Developing speed Organization
Inner Dynamics of Writing	Patience Blank page paralysis Focus Self-criticism Moving from planning to writing	Inner Dynamics of Writing	No mentions of concerns in this category post-course
Scientific Literacy	Understanding big picture Scope of work Integrating themes and details	Scientific Literacy	Explaining to someone outside my field Ambition vs. realistic project goals Broad vs. general language
EXPECTATIONS/OUTCOMES			
*Expectations Themes*	*Examples*	*Outcomes Themes*	*Examples*
Preparation for Future Work	F30/F31 grants Comprehensive exam	Increased Confidence in Grant Writing	Appreciation for practice and iterative improvement Increased awareness of my own quirks Increased toolkit for self-revision
Developing New Skills/Ways of Thinking	New writing skills Organizing information Critical thinking	Increased Skills in Grant Writing	How to start Writing clearly Writing for varied audiences
Acquiring New Knowledge	Grant vocabulary/processes Deeper knowledge of field	Increased Knowledge of Grant-Specific Elements	Components of the grant Linkage between sections Differences between writing grants and manuscripts
		Increased Willingness to Seek Feedback on Writing	Recognizes value of feedback Better at overcoming my own resistance to criticism Value of feedback outside of my field Asking specific questions
		Improved Ability to Provide Feedback	Have language to discuss writing, grammar, etc. Knowledge of grant norms informs feedback Self-efficacy supported by my feedback being consistent with other reviewers
		More Writing Enjoyment	Reduced writing anxiety/writer’s block Reduced procrastination

n = 35 (100% response rate).

Post-course interview commentary corroborated data from anonymous surveys. For example, of the 32 students interviewed, 100% felt that their knowledge of how to write a grant had improved, and most (94%) reported that their confidence in grant writing had grown (Table 4). Participants described acquiring knowledge of grant-specific elements (e.g., components required, interconnections within a grant), as well as improving their awareness of writing processes (e.g., how to start, time management) and their skills in technical language (style, word choice, expert and novice audiences). Learners spoke of an increase in their confidence because they became more aware of their own writing style, quirks, and preferences. They described writing techniques and strategies that stuck with them and had become individual norms: structuring time and defining tasks for writing work; writing in waves; and starting with grant writing templates to generate a bulleted outline and ensure that the big picture is apparent from the beginning.

Post-course interviews also indicated that most students (67%) were more willing to seek feedback than they were before the course, and that nearly all (97%) felt that their ability to provide feedback had improved (Table 4). Acquiring knowledge of good writing mechanics and the structure and function necessary for grant writing enabled them to be more specific in the feedback they provided. Also, providing feedback within the context of a writing group allowed the students to see which of their contributions were consistent with those of others, building confidence in this skill. They recognized the high value of peer feedback and were less resistant to criticism. Many students noted that feedback from readers outside their area of scientific expertise was particularly valuable. These latter findings differ from students’ views expressed in other grant writing courses [19], suggesting that feedback in the context of small writing groups might successfully increase perceptions of the value of peer feedback.

When students were asked which aspects of GWB were most enjoyable (Table 4), most (67%) mentioned the interactive feedback sessions. In addition, students stated that they appreciated the following: 1) developing a Specific Aims page from start to finish and having confidence in it; 2) learning about each other’s research projects; 3) discussing examples of sections from previously submitted fellowship applications in class; 4) having templates to use when starting to write a Specific Aims page and other sections of grants; 5) receiving validation that writing is intimidating; 6) brainstorming potential solutions to overcome barriers; 7) developing confidence as a reviewer, especially when the facilitator agreed with their point and built on it; and 8) hearing from faculty reviewers with diverse backgrounds and opinions. One student commented that GWB “was the highlight of my week!”

Post-course interviews also uncovered common, enduring fears about the uncertainties of grant writing. First, many students expressed concerns about their readiness to complete grant proposal sections other than the Specific Aims page (e.g., the Research Strategy, Biographical Sketch, Training Plan). These concerns reflect that these sections were discussed but were not extensively critiqued. Second, students often said that they would miss the routine peer feedback and the structure provided in the course as they continued to work on their fellowship applications, noting they would need to establish their own deadlines and writing routines. These concerns were allayed by providing students with information about additional resources that would be available to them after GWB ended.

## Discussion

The results of this study demonstrate that providing formal course-based training in grant writing to graduate students leads to consistent positive outcomes. These include increased knowledge and skills in grant writing fundamentals; an expanded repertoire of strategies for organizing and managing a grant writing project; and increased willingness to seek and provide feedback on writing, even outside of their own area of expertise.

A comparison of students’ self-identified pre- and post-course strengths reveals that they had developed more specific and action-based language to describe their strengths (e.g., pre-course: “organization” versus post-course: “defining task lists, scheduling time, drafting in bullet points”). Interestingly, no student identified giving and receiving feedback as a writing-related strength before the course, but most commented on their capacity to provide and receive feedback as nascent strengths after the course. Students had also developed more specific language to describe their weaknesses, likely informed by the language they acquired in the course. Notably, although many students described weaknesses related to self-doubt, anxiety, and difficulty with motivation before taking the course, none identified these as current weaknesses afterwards. The structures and scaffolds provided in this course likely helped create a path to overcome internal challenges. Finally, when students talked about the difficulty of explaining the big picture of their work after the course, they most often referred to the needs of the reader and the importance of explaining their science to a non-specialist audience. This was rarely mentioned as a development area before students took the course.

Pre-course interviews provided students with an understanding of course expectations. Post-course interviews provided an opportunity to explore whether expectations were met, as well as to determine whether students experienced any unexpected outcomes. Strikingly, the expectations expressed in pre-course interviews were frequently described as outcomes during post-course interviews. Often the language was nuanced, reflecting new perspectives that students gained. Several unexpected outcomes were also described, including a reduction in the emotional challenges of writing (e.g., anxiety), development of confidence around writing work, and increased willingness and capacity to both give and receive feedback.

One metric for judging the success of GWB is the numbers of fellowship submissions and awards, a common benchmark among grant writing courses [4,10,19,20]. As noted above, MSTP students are encouraged but not required to submit a fellowship application. This decision reflects the diversity of research topics, the structure of the graduate programs (which can make timely submission a challenge), and the fact that only a subset of relevant funding agencies offer fellowship grants. For example, not all NIH Institutes participate in the F30 NRSA funding mechanism. Thus, the scope of research areas covered by these awards is somewhat limited. F31 applications are another option (all PhD students are eligible). Private foundations provide additional funding opportunities, but these do not broaden the options substantially. Notwithstanding these limitations, most GWB students who took the course from 2015 to 2021 (77%, n = 66) submitted predoctoral fellowship applications (Table 1); this represents an increase over the number of submissions from MSTP cohorts over the prior 7-year period (2008 to 2014; 62%, n = 66). Funding rates depend upon multiple factors, including the NIH Institute for submission. Our student success rates are high (49%; Table 1) and exceed the overall F30 success rate at NIH over the same period (39%; [21]). Anecdotally, completion of GWB also improved outcomes of our students’ comprehensive exams, which often take the form of an NIH predoctoral fellowship or small research (e.g., R21) proposal. Notably, one of the GWB instructors was contacted by the chair of an MSTP student’s comprehensive exam committee because he was “impressed by the clarity and reader-friendliness of her [the student’s] written proposal. And when [the student was] asked about how she learned how to write, she mentioned the MSTP GWB class.” Perhaps the most gratifying review of GWB came from a student who stated, “I found grants really intimidating before this class, but now feel empowered, even excited, about grant writing.”

Limitations to this study relate mainly to study design. First, no concurrent control group was included. Second, because this is an observational study, it is not possible to definitively claim cause-effect, i.e., although successful grant funding is a useful indicator of writing quality, a funded grant is also a product of external forces that are outside the control of the student/writer. One strength of the study is inclusion of multiple cohorts in the overall data set, yet the fact that the course evolved during the study period makes it impossible to know the individual impacts of each incremental change in the program.

One observation that might be considered a limitation of GWB is that most students continue to feel insufficiently prepared to write a full proposal. A continued need for writing instruction aligns with the increased awareness across disciplines that writing in subject-matter courses needs to occur routinely throughout training [22,23]. This need was further supported by a senior MSTP student’s observation that for her the “rest” of the grant came together in a later grant writing course provided in her graduate training program (University of Iowa Interdisciplinary Graduate Program in Immunology). Continued formal training in writing is possible for many students, as other University of Iowa graduate programs also offer courses in grant writing. Notably, a grant writing program for MSTP students at the University of Alabama described pairing a grant writing course early in a student’s plan of study with a seminar series that provides both advice on how to write grant sections and the opportunity to receive feedback on drafts [4]. Similarly, we encourage students to build on what they learn in GWB by continuing to both meet with members of their writing groups and work with the editors on the instructional team when writing new sections of their grants. Notably, the skills needed in preparing grants and research articles differ, emphasizing that it is important for students to continue to seek advice concerning scientific writing from research mentors and others in the research community. Thus, we provide the students with information about university resources, including editing services.

The benefits of GWB are not limited to the MSTP. We have shared our course materials with instructors of other PhD training programs to encourage submission of NIH F31 applications, and this has had a positive impact on their courses. GWB also represents an excellent example of how program evaluation contributes to a healthy education ecosystem. Evaluation data have provided insight into the impact of the course and encouraged its iterative evolution. Moreover, participation in the evaluation process has enabled students to become engaged with the educational process and the leadership team, a step that is necessary for developing a feeling of belonging and value in academic culture.

## Conclusions: Return on investment

The findings presented here illustrate the value of a dedicated writing course to “jump-start” students’ grant writing and prepare them for a career that will require extensive scientific writing. In particular, GWB supports the value of using a grant application as a guiding structure in teaching scientific writing. Our course evaluations highlight the value of using a flipped-classroom model that allows more time for active learning. Aspects of this model that were especially effective were the initial requirement for students to evaluate example sections from previously submitted grants and the subsequent requirement to participate in intensive, iterative rounds of critiquing the students’ own writing samples. Notably, the most popular outcomes reported by the students were their increased confidence in providing feedback and their newfound appreciation for feedback from a broad audience.

The GWB course represents a substantial investment of both student and instructor time. We estimate that it requires at least two skilled facilitators with complementary expertise in scientific writing and experimental design. Nevertheless, the positive outcomes are clear, and they strongly support the use of similar approaches by other science training programs.

## Supporting information

S1 Appendix Pre-course interview questions. (DOCX)

S2 Appendix Post-course interview questions. (DOCX)

S1 Table Evolution of GWB course structure from 2015 to 2023. (DOCX)

## References

[1] Cable CT, Boyer D, Colbert CY, Boyer EW. The writing retreat: a high-yield clinical faculty development opportunity in academic writing. J Grad Med Educ. 2013;5(2):299–302. Epub 2014/01/10. doi: 10.4300/JGME-D-12-00159.1 . 24404277 PMC3693698

[2] Arum R, Roksa J. Academically adrift: limited learning on college campuses. Chicago, IL: University of Chicago Press; 2011.

[3] Verderame MF, Freedman VH, Kozlowski LM, McCormack WT. Competency-based assessment for the training of PhD students and early-career scientists. Elife. 2018;7. Epub 2018/06/01. doi: 10.7554/eLife.34801 . 29848440 PMC6002247

[4] Souder JP, Pepin ME, Seay RL, Lorenz RG, Geisler WM, Yacoubian T. A novel curricular framework to develop grant writing skills among MD-PhD students. J Clin Transl Sci. 2022;6(1):e54. Epub 2022/06/04. doi: 10.1017/cts.2022.384 . 35656336 PMC9120620

[5] Chepp V, Baker C, Kostiha S, Smith JD. The Molecular Medicine PhD program alumni perceptions of career preparedness. PLoS One. 2022;17(11):e0275996. Epub 2022/11/18. doi: 10.1371/journal.pone.0275996 . 36395255 PMC9671420

[6] Burgess A, McGregor D. Peer teacher training for health professional students: a systematic review of formal programs. BMC Med Educ. 2018;18(1):263. Epub 2018/11/18. doi: 10.1186/s12909-018-1356-2 30442139 PMC6238310

[7] Knowles MS, Holton EF, Swanson RA. The adult learner: the definitive classic in adult education and human resource development. Burlington, MA: Elsevier; 2005.

[8] Professionals. NOoRD. Grant Writing Courses. https://www.nordp.org/assets/resources-docs/grant_writing_courses.pdf.

[9] Walsh B, Bonner D, Springer V, Lalasz C, Ives B. Grant writing courses in the United States: A descriptive review of syllabi and factors that influence instructor choice of texts. College Teaching. 2013;61(2):74–81. doi: 10.1080/87567555.2012.741080

[10] Eisennberg T. Teaching successful grant writing to psychology graduate students. Teaching of Psychology. 2003;30(4):328–30.

[11] Kahn RA, Conn GL, Pavlath GK, Corbett AH. Use of a Grant Writing Class in Training PhD Students. Traffic. 2016;17(7):803–14. Epub 2016/04/12. doi: 10.1111/tra.12398 . 27061800

[12] Gantwerker EA, Lee GS. Principles of Adult Learning: Tips for the Pediatric Otolaryngologist. Otolaryngol Clin North Am. 2022;55(6):1311–20. Epub 2022/11/13. doi: 10.1016/j.otc.2022.07.009 . 36371143

[13] Moore AL, Chaisson NF. Development of a Scientific Writing Course to Increase Fellow Scholarship. ATS Sch. 2022;3(3):390–8. Epub 2022/11/01. doi: 10.34197/ats-scholar.2022-0023PS . 36312809 PMC9590585

[14] Chen F, Lui AM, Martinelli SM. A systematic review of the effectiveness of flipped classrooms in medical education. Med Educ. 2017;51(6):585–97. Epub 2017/05/11. doi: 10.1111/medu.13272 . 28488303

[15] Robertson JD, Russell SW, Morrison DC. The Grant Application Writer’s Workbook; National Institute of Health. Buelton, CA: Grant Writers’ Seminars and Workshops, LLC; 2019.

[16] AtKisson MS. The Handbook for Planning and Writing Successful Grant Proposals2021.

[17] Grant A. How to love criticism 2018. https://www.ted.com/talks/worklife_with_adam_grant_how_to_love_criticism/transcript?language=en.

[18] Kellems KC, Monson C, Mitchell J. Maximizing Grant Proposal Writing for Success Using “The Baseline Logic Model”. https://www.nordp.org/assets/RDConf2019/presentations/nordp-2019_conf-monson.pdf.

[19] Leak RK, O’Donnell LA, Surratt CK. Teaching Pharmacology Graduate Students how to Write an NIH Grant Application. Am J Pharm Educ. 2015;79(9):138. Epub 2015/11/25. doi: 10.5688/ajpe799138 . 28435165 PMC5394916

[20] Weber-Main AM, McGee R, Eide Boman K, Hemming J, Hall M, Unold T, et al. Grant application outcomes for biomedical researchers who participated in the National Research Mentoring Network’s Grant Writing Coaching Programs. PLoS One. 2020;15(11):e0241851. Epub 2020/11/10. doi: 10.1371/journal.pone.0241851 . 33166315 PMC7652313

[21] Success Rates RePORT: Fellowships (Fs): Competing applications a, success rates and funding, by activity code and Institute Center. 2013–2023. https://report.nih.gov/funding/nih-budget-and-spending-data-past-fiscal-years/success-rates.

[22] Melzer D, Bean J, editors. Engaging Ideas: The Professor’s Guide to Integrating Writing, Critical Thinking, and Active Learning in the Classroom. ProQuest Ebook Central: John Wiley & Sons; 2021.

[23] Writing Across the Curriculum (WAC) Clearinghouse. https://wac.colostate.edu/repository/resources/teaching/intro/include/.

