# Supplementary material for: Granting access: Development of a formal course to demystify and promote predoctoral fellowship applications for graduate students
Source: PLoS One. 2024 Apr 26;19(4):e0301480. doi: 10.1371/journal.pone.0301480 (PMC11051599; doi:10.1371/journal.pone.0301480)
Supplement: S1 Appendix — (DOCX) [file pone.0301480.s001.docx]

Supplementary Materials for

Granting Access: Development of a formal course

to demystify and promote

predoctoral fellowship applications for PhD students

Pamela K. Geyer^1,2,^* , Darren S. Hoffman^2,3^, Jennifer Y. Barr^4^, Heather A. Widmayer^4^,

Christine M. Blaumueller^4,^*

^1^Department of Biochemistry and Molecular Biology

^2^Medical Scientist Training Program

^3^Department of Anatomy and Cell Biology

^4^Scientific Editing and Research Communication Core

Carver College of Medicine, University of Iowa, Iowa City, IA 52242

* Co-Corresponding Authors:

Pamela Geyer, Department of Biochemistry

3135E MERF, University of Iowa

Iowa City, IA 52242, USA

Tel. 319 335-6953

FAX. 319 384-4770

email: [pamela-geyer@uiowa.edu](mailto:pamela-geyer@uiowa.edu)

Christine M. Blaumueller, Scientific Editing and Research Communication Core

134 CMAB, University of Iowa

Iowa City, IA 52242, USA

Tel. 319 335-8095

email: [christine-blaumueller@uiowa.edu](mailto:christine-blaumueller@uiowa.edu)

**Appendix I Pre-course interview questions**

1. What problem and/or specific gap in knowledge does your project address?
2. How will your project move the field forward/what is the expected broader impact of your work?
3. Have you written a Specific Aims page before?
4. Do you have experience writing a grant proposal?
5. Have you written a research article, or parts of a research article, before?
6. Do you have experience providing feedback on a research article? This could include anything from contributing to a review for a journal article to providing feedback on a paper by a peer or lab mate.
7. Based on your past experience doing scientific writing, what do you feel your strengths are?
8. From your past experience doing scientific writing, what have you struggled with?
9. What are your expectations for the course? What do you hope to get out of it?
