# Supplementary material for: Granting access: Development of a formal course to demystify and promote predoctoral fellowship applications for graduate students
Source: PLoS One. 2024 Apr 26;19(4):e0301480. doi: 10.1371/journal.pone.0301480 (PMC11051599; doi:10.1371/journal.pone.0301480)
Supplement: S2 Appendix — (DOCX) [file pone.0301480.s002.docx]

**Appendix II Post-course interview questions**

1. How has your knowledge of how to write a grant improved?
2. Has your ability to present your ideas on a Specific Aims page improved (e.g., the gap or its importance, the objective, the aims, etc.)?
3. Have you moved forward significantly toward writing a grant for submission to a funding agency?
   1. Do you feel like you have a Specific Aims page that is close to usable for a submission?
   2. Are you more confident about writing the rest of the grant than you were in January?
4. Has your willingness to seek out feedback on your writing changed?
5. Has your ability to provide feedback on someone else’s writing improved?
6. Have your writing skills improved? If so, how?
7. Have you overcome problems that you previously experienced in writing?
   1. What kind of mechanisms have you established to help with this?
   2. What do you still need to work on?
8. How effective did you find the time set aside in class to work on writing different sections of the fellowship proposal?
9. Have your expectations of the course been met?
10. What did you enjoy most about the course?
11. What was your least favorite part of the course?
