# Supplementary material for: Granting access: Development of a formal course to demystify and promote predoctoral fellowship applications for graduate students
Source: PLoS One. 2024 Apr 26;19(4):e0301480. doi: 10.1371/journal.pone.0301480 (PMC11051599; doi:10.1371/journal.pone.0301480)
Supplement: S1 Table — (DOCX) [file pone.0301480.s003.docx]

**Supplementary Table 1. Evolution of GWB course structure from 2015 to 2023**

| Year | Number of Students | Timing and format | Writing groups | Changes made (the following year) based on feedback from this group |
| --- | --- | --- | --- | --- |
| 2015 | 10 | Summer, 1 session per week over 3 months; wrote and received feedback on multiple grant sections; in-person | Small writing groups (3 groups of 3–4 students); unfacilitated but overseen by 2 instructors: faculty and editor | Time changed to Spring semester and course shortened; critique sessions switched to large-group setting; only Specific Aims page critiqued |
| 2016 | 9 | Spring semester, 2 sessions per week for 7 weeks; in-person | Large writing group (1 group of 9 students); facilitated by 2 instructors (faculty and editor) |  |
| 2017 | 11 | Spring semester, 2 sessions per week for 5 weeks; in-person | Large writing group (1 group of 11 students); facilitated by two instructors (faculty and editor) on rotating basis | Expanded course to a semester; returned to small writing groups |
| 2018 | 9 | Spring semester, 1 session per week for 11 weeks; in-person | Small writing groups (3 groups of 3 students); facilitated by 3 instructors (faculty and editors) on a rotating basis | – |
| 2019 | 11 | Spring semester, 1 session per week for 11 weeks; in-person | Small writing groups (3 groups of 3 students); facilitated by 3 instructors (faculty and editors) on a rotating basis | Introduced pre- and post-course interviews; had the same facilitators for writing-group sessions; adopted fully flipped classroom, with lectures studied prior to session (PPT or PDF file) |
| 2020 | 6 | Spring semester, 1 session per week for 11 weeks; in-person first half of semester and then Zoom (due to pandemic) | Small writing groups (2 groups of 3 students); facilitated by 2 instructors (editors), with third instructor (faculty) switching between groups | Fully flipped classroom approach enhanced by availability of recordings of lectures |
| 2021 | 10 | Spring semester, 1 session per week for 11 weeks; Zoom | Small writing groups (3 groups of 3–4 students); facilitated by 3 instructors (editors), with fourth instructor (faculty) switching between groups | Introduction of writing/ brainstorming time to each "Workshop" session to get start on all grant sections of application |
| 2022 | 8 | Spring semester, 1 session per week for 11 weeks; Zoom | Small writing groups (2 groups of 4 students); facilitated by instructors (editors), with fourth instructor (faculty) switching between groups | – |
| 2023 | 8 | Spring semester, 1 session per week for 11 weeks; Zoom | Small writing groups (2 groups of 4 students); facilitated by instructors (editors), with fourth instructor (faculty) switching between groups | – |
